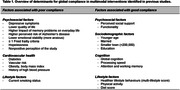# Measuring and reporting adherence in multimodal interventions to prevent cognitive decline

**DOI:** 10.1002/alz.092137

**Published:** 2025-01-09

**Authors:** Natalia Soldevila‐Domenech, Amaia Ayala‐García, Laura Forcano, Mariagnese Barbera, Jenni Lehtisalo, Tiia Ngandu, Alina Solomon, Rafael de la Torre

**Affiliations:** ^1^ Integrative Pharmacology and Systems Neurosciences Research Group, Neurosciences Research Program, Hospital del Mar Research Institute, Barcelona Spain; ^2^ University of Eastern Finland, Kuopio Finland; ^3^ Population Health Unit Finnish Institute for Health and Welfare, Helsinki Finland; ^4^ Population Health Unit, Finnish Institute for Health and Welfare, Helsinki Finland; ^5^ Department of Clinical Medicine/Neurology, University of Eastern Finland, Kuopio, Finland, Kuopio Finland

## Abstract

**Background:**

Adhering to lifestyle‐based multimodal interventions to prevent cognitive decline is crucial for their success, but limited evidence exists on determinants of adherence. This gap may stem from inconsistent adherence measurement and reporting across studies. Additionally, uncertainties persist regarding the optimal intervention intensity needed for meaningful cognitive benefits. Addressing these gaps is vital for conducting comparative effectiveness research and for the successful implementation of precision prevention interventions. This study aims to present current evidence on determinants of adherence to multimodal interventions, and discuss how to harmonize the assessment of adherence and the reporting of multimodal interventions intensity in World‐Wide FINGER studies.

**Method:**

To identify determinants of global adherence to multimodal interventions, we conducted a systematic search on PubMed and employed snowball methods, involving the pursuit of references within references and electronic citation tracking.

**Result:**

As of January 2024, five studies quantitatively explored factors influencing global compliance or participation with multimodal interventions’ activity programs (Table 1), covering sociodemographic, lifestyle, psychological, cardiovascular, and cognitive factors. However, there was high heterogeneity in compliance reporting; some studies used a 66% completion criterion, while others employed different thresholds (e.g., 40‐50% participation in at least 50% of domains). We argue that using an arbitrary percentage as a compliance cut‐off might not be informative as it depends on the dose delivered and the length of follow‐up; i.e., the intensity of the intervention. We suggest reporting average participation in each intervention component to allow for better cross‐trial comparisons. Additionally, we suggest calculating expected intervention intensity as the ratio between the prescribed dose (number of pre‐specified sessions) and the length (months). Observed intensity could be estimated with the same formula, but correcting the prescribed dose by the average compliance to each intervention component. Lastly, we suggest rating group and individual sessions differently to estimate intervention compliance, and emphasize that global adherence should encompass both compliance and lifestyle change.

**Conclusion:**

Harmonizing adherence reporting in World‐Wide FINGER studies will enhance comprehension of how intervention intensity impacts efficacy. It will also enable pooled analyses to explore adherence determinants, offering valuable insights for designing future precision studies for dementia prevention.